# National trends in incidence and outcomes of abdominal aortic aneurysm among elderly type 2 diabetic and non-diabetic patients in Spain (2003–2012)

**DOI:** 10.1186/s12933-015-0216-1

**Published:** 2015-05-07

**Authors:** Ana Lopez-de-Andrés, Isabel Jiménez-Trujillo, Rodrigo Jiménez-García, Valentín Hernández-Barrera, José Mª de Miguel-Yanes, Manuel Méndez-Bailón, Napoleón Perez-Farinos, Miguel Ángel Salinero-Fort, Pilar Carrasco-Garrido

**Affiliations:** Preventive Medicine and Public Health Teaching and Research Unit, Health Sciences Faculty, Rey Juan Carlos University, Avda. de Atenas s/n, 28922 Alcorcon, Comunidad deMadrid Spain; Medicine Department, Hospital Gregorio Marañon, Comunidad de Madrid, Spain; Medicine Department, Hospital Clínico San Carlos, Madrid, Comunidad de Madrid Spain; Health Security Agency, Ministry of Health, Madrid, Comunidad de Madrid Spain; Dirección Técnica de Docencia e Investigación, Gerencia Atención Primaria, Madrid, Comunidad de Madrid Spain

**Keywords:** Type 2 diabetes, Abdominal aortic aneurysm, Elderly, Hospitalization, Length of stay, In-hospital mortality

## Abstract

**Background:**

This study aims to describe trends in the rate of abdominal aortic aneurysm (AAA) and use of open surgery repair (OSR) and endovascular aneurysm repair (EVAR) in elderly patients with and without type 2 diabetes in Spain, 2003–2012.

**Methods:**

We select all patients with a discharge of AAA using national hospital discharge data. Discharges were grouped by diabetes status: type 2 diabetes and no diabetes. In both groups OSR and EVAR were identified. The incidence of discharges attributed to AAA were calculated overall and stratified by diabetes status and year. We calculated length of stay (LOHS) and in-hospital mortality (IHM). Use of OSR and EVAR were calculated stratified by diabetes status. Multivariate analysis was adjusted by age, sex, year, smoking habit and comorbidity.

**Results:**

From 2003 to 2012, 115,020 discharges with AAA were identified. The mean age was 74.91 years and 16.7% suffered type 2 diabetes. Rates of discharges due to AAA increased significantly in diabetic patients (50.09 in 2003 to 78.23 cases per 100,000 in 2012) and non diabetic subjects (69.24 to 78.66). The incidences were higher among those without than those with diabetes in all the years studied.

The proportion of patients that underwent EVAR increased for both groups of patients and the open repair decreased. After multivariate analysis we found that LOHS and IHM have improved over the study period and diabetic patients had lower IHM than those without diabetes (OR 0.81; 95%CI 0.76-0.85).

**Conclusions:**

Incidence rates were higher in non-diabetic patients. For diabetic and non diabetic patients the use of EVAR has increased and open repair seems to be decreasing. IHM and LOHS have improved from 2003 to 2012. Patients with diabetes had significantly lower mortality.

## Background

Diabetes is a risk factor for peripheral, coronary, and cerebrovascular disease. However different studies have associated diabetes with the reduced risk of abdominal aortic aneurysm (AAA) [1-4]. A recent meta-analysis reported that patients with diabetes have a lower prevalence of AAA and they show a decreased risk of developing new AAA or enlarging their AAA compared with non-diabetics [5].

The primary risk associated with AAA is rupture, which may occur suddenly and without symptoms and is often fatal. An estimated 59% to 83% of patients with AAA rupture die before hospitalization and operative mortality (in hospital or 30-day) is approximately 40%. Almost all deaths from rupture occur after age 65 years, and most deaths in women occur after age 80 years [6,7].

Since its introduction in 1991, endovascular aneurism repair (EVAR) has significantly extended [8]. EVAR is an alternative to open surgery repair (OSR), especially in patients for whom open surgery poses a considerable risk due to coexisting medical conditions, including patients with diabetes [9]. Whether diabetes is a risk factor for mortality and morbidity after AAA repair is controversial. In 2005, Leurs *et al.* have shown that people with diabetes have a significantly higher early mortality rate as well as a higher incidence of device-related complications compared with non-diabetics following endovascular AAA repair [10]. An increased perioperative morbidity and mortality risk for people with diabetes undergoing aortic surgery, however, is not universally accepted. There have been studies that have shown that diabetes is not associated with significantly worse major outcomes following AAA repair [11,12]. Indeed, Hughes *et al.* reported that following open, elective, infra-renal AAA repair, diabetes is not associated with an increased risk of mortality compared with non-diabetics (OR 1.4, 95%CI 0.68-2.71) [13].

The prevalence of AAA in Spain has been reported in previous investigations [14-18]. However, most studies included small samples and were conducted on primary health care centers or hospital services using ultrasonography as the diagnosis method. The prevalence observed for the 65–75 year age group ranged from 3% to 5% [14-18]. In Spain there is no population based screening program for AAA and the Medical Societies recommend screening for AAA with ultrasonography in men aged 65 to 75 years who have ever smoked [16].

To our knowledge, no previous studies have investigated national trends in the use and outcomes of open and endovascular AAA repair in diabetic and non diabetic patients in Spain.

In this study, we used national hospital discharge data to examine trends in the incidence of AAA among hospitalized elderly patients with and without type 2 diabetes between 2003 and 2012 in Spain. In particular, we analyzed trends in the use of open and endovascular AAA repair, patient comorbidities, and in-hospital outcomes such as in-hospital mortality (IHM) and length of hospital stay (LOHS).

## Methods

This retrospective, observational study was conducted using the Spanish National Hospital Database (CMBD*, Conjunto Minimo Básico de Datos*). This database is managed by the Spanish Ministry of Health, Social Services and Equality and compiles all public and private hospital data, hence covering more than 95% of hospital discharges [19]. The CMBD includes patient variables (sex, date of birth), admission date, discharge date, up to 14 discharge diagnoses, and up to 20 procedures performed during the hospital stay. The Spanish Ministry of Health, Social Services and Equality sets standards for record-keeping and performs periodic audits [19]. Data collected between January 1, 2003 and December 31, 2012 were analyzed.

Disease and procedure criteria were defined according to the International Classification of Diseases-Ninth Revision, Clinical Modification (ICD-9-CM), which is used in the Spanish CMBD.

We selected discharges for subjects whose medical diagnosis included AAA codes according to the ICD-9-CM: 441.3; 441.4; 441.5 in any diagnosis field. We only included subjects aged 50 years or over because the very low incidence of AAA is in the population under this age [3].

We identified open AAA repairs and endovascular AAA repairs using ICD-9-CM procedures codes, 38.44 and 39.71, respectively.

Discharges were grouped by diabetes status as follows: type 2 diabetes (ICD-9-CM codes: 250.x0; 250.*x*2) and no diabetes. Patients with type 1 diabetes (ICD-9-MC codes: 250.x1; 250.x3) were excluded.

Clinical characteristics included information on overall comorbidity at the time of diagnosis, which was assessed by calculating the Charlson comorbidity index (CCI). The index applies to 17 disease categories, the scores of which are added to obtain an overall score for each patient [20]. We divided patients into three categories: low index, which corresponds to patients with no previously recorded disease or with one disease category; medium index, patients with two categories; and high index, patients with three or more disease categories. To calculate the CCI we used 16 disease categories, excluding diabetes, as described by Thomsen RW *et al.* [21].

Information on smoking was identified using ICD-9-CM codes: 305 and V1582.

The mean LOHS and the proportion of patients that died during the hospital admission (IHM) were also estimated for each year studied.

Before the analysis was conducted we checked the database for any missing data on the following variables: Sex, Date of birth, Admission date, Discharge date and if the patient died during the hospitalization. If any of these variables were missing the record was deleted for the analysis. As all the databases pass a quality control at the Ministry of Health before are sent to the investigators we had to discharge under 0.1% of records.

### Statistical analysis

To assess time trends, rates of AAA discharges and open and endovascular repairs for type 2 diabetes and non-diabetic patients were calculated in terms of 100,000 inhabitants. We calculated yearly diabetes-specific incidence rates dividing the number of cases per year, sex, and age group by the corresponding number of people in that population group, using age- and sex-adjusted estimated prevalence of diabetes obtained from National Health Surveys conducted in 2003/4, 2006/7, 2009/10 and 2011/12 and data from Di@bet.es Study [22,23]. We also calculated the yearly age- and sex-specific incidence rates for non-diabetic patients dividing the number of cases per year, sex, and age group by the corresponding number of people in that population group (excluding those with type 2 diabetes), according to data from the Spanish National Institute of Statistics, as reported on December 31 of each year [24].

A descriptive statistical analysis was performed for all continuous variables and categories by stratifying discharges for AAA, open and endovascular repairs according to diabetes status. Variables are shown as proportions, means with standard deviations or medians with interquartile ranges (LOHS). Bivariate analyses of variables according to year was using *χ*2 linear trend analysis (proportions), ANOVA (means) and Kruskall-Wallis test (medians), as appropriate.

In order to test the time trend in the incidence due to abdominal aortic aneurysm, we fitted separate Poisson regression models for patients with and without type 2 diabetes, using year of discharge, sex, age, CCI, smoking and type of repair as independent variables. A global model including the same variables and diabetes status was also conducted to assess the adjusted effect of diabetes in the incidence.

For IHM, logistic regression analyses were performed with mortality as a binary outcome using the same independent variables for those with and without diabetes and for the entire population to assess the influence of diabetes on IHM. Statistical analyses were performed using Stata version 10.1 (Stata, College Station, Texas, USA). Statistical significance was set at p < 0.05 (2-tailed).

### Ethical aspects

Data confidentiality was maintained at all times in accordance with Spanish legislation. Patient identifiers were deleted before the database was provided to the authors in order to maintain patient anonymity. It is not possible to identify patients on individual levels, either in this article or in the database. Given the anonymous and mandatory nature of the dataset, it was not necessary to obtain informed consent. The study protocol was approved by the ethics committee of the Universidad Rey Juan Carlos.

## Results

We identified a total of 115,020 discharges of patients admitted with AAA in Spain from 2003 to 2012. Patients with type 2 diabetes accounted for 16.7% of total (19,232); mean age was 74.75 years (SD, 8.86 years) and 93.3% were men. In patients without diabetes mean age was 74.95 years (SD, 8.86 years) and 91.9% were men.

Patients with type 2 diabetes had significantly higher CCI values compared with those without diabetes (72% vs. 65.9% with two or more coexisting conditions, respectively).

We found that the proportion of patients who smoked was 33.6% in patients with type 2 diabetes and 34.3% in those without diabetes.

**Figure 1:**
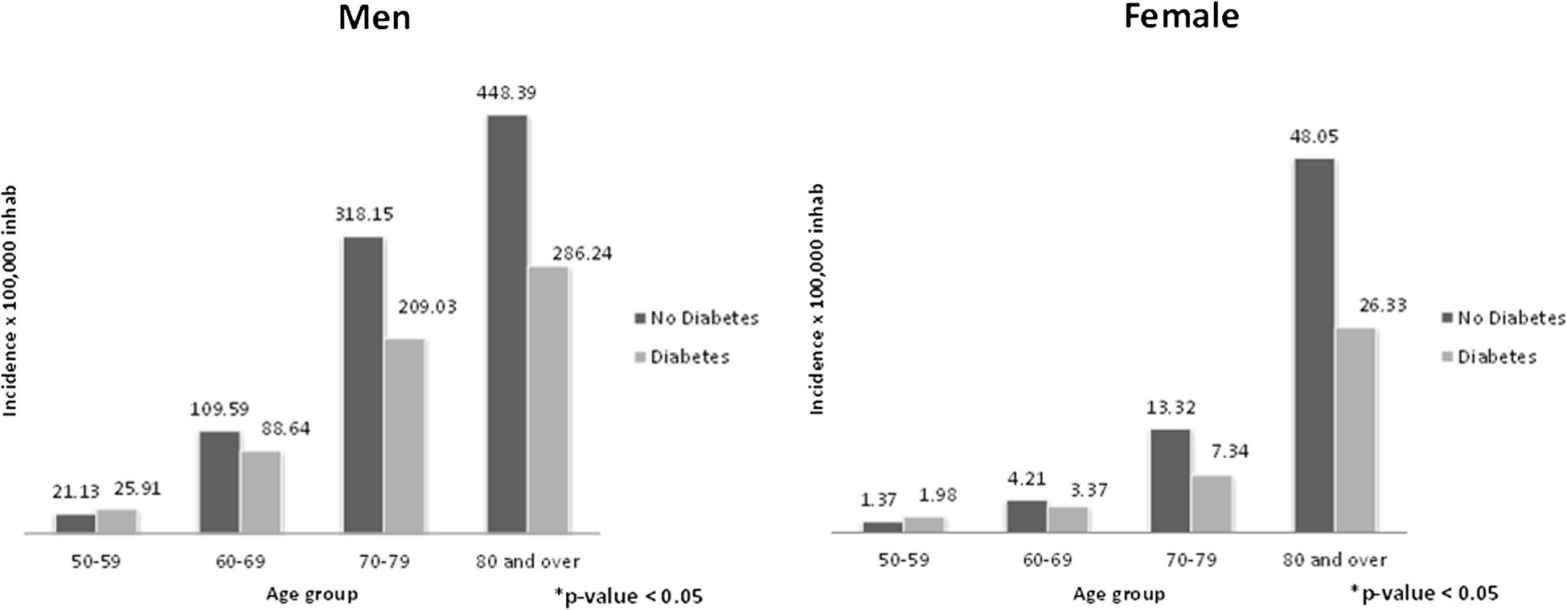
Incidence of abdominal aortic aneurysm among men and women with and without type 2 diabetes by age group.

Shown in Figure 1 are the cumulative incidences of discharges for AAA according to age group for men and women with and without diabetes along the entire study period (2003–2012). The incidences were significantly higher among men and women without diabetes aged 70–79 and 80 years or over than in patients with type 2 diabetes.

Table 1 shows the annual hospital discharges rates, clinical characteristic and outcomes for patients with an AAA discharge diagnosis according to diabetes status from 2003 to 2012. The cumulative incidence of discharges due to AAA in patients with diabetes increases significantly from 50.09 cases per 100,000 inhabitants in 2003 to 78.23 cases in 2012. In patients without diabetes, the incidence increased significantly from 69.24 cases per 100,000 inhabitants in 2003 to 78.66 cases in 2012. The incidences were higher among those without than those with diabetes in all the years studied.

**Table 1:** **Incidences, clinical characteristics and outcomes of hospital discharges due to abdominal aortic aneurysm among patients with and without type 2 diabetes in Spain, 2003-2012**

	**2003**	**2004**	**2005**	**2006**	**2007**	**2008**	**2009**	**2010**	**2011**	**2012**	**Total**
No Diabetes											
N	8157	8388	8857	9002	9305	9508	10208	10724	10871	10768	95788
Incidence*	69.24	71.2	72.53	71.2	73.6	73.25	76.64	80.51	79.41	78.66	74.80
Age, mean (SD)*	74.05 (8.59)	74.06 (8.61)	74.27 (8.7)	74.44 (8.71)	74.76 (8.73)	75.08 (8.8)	75.35 (8.93)	75.43 (9.08)	75.58 (8.97)	75.88 (9.08)	74.95 (8.86)
Male, n (%)*	7499 (91.93)	7747 (92.36)	8193 (92.5)	8334 (92.58)	8622 (92.66)	8775 (92.29)	9315 (91.25)	9839 (91.75)	9932 (91.36)	9808 (91.08)	88064 (91.94)
CCI 0–1, n (%)*	3083 (37.8)	2998 (35.74)	3166 (35.75)	3280 (36.44)	3286 (35.31)	3181 (33.46)	3342 (32.74)	3467 (32.33)	3468 (31.9)	3387 (31.45)	32658 (34.09)
CCI 2, n (%)	2808 (34.42)	2944 (35.1)	3091 (34.9)	3148 (34.97)	3244 (34.86)	3433 (36.11)	3595 (35.22)	3898 (36.35)	3827 (35.2)	3706 (34.42)	33694 (35.18)
CCI ≥3, n (%)	2266 (27.78)	2446 (29.16)	2600 (29.36)	2574 (28.59)	2775 (29.82)	2894 (30.44)	3271 (32.04)	3359 (31.32)	3576 (32.89)	3675 (34.13)	29436 (30.73)
Smoking*	2615 (32.06)	2789 (33.25)	2960 (33.42)	2891 (32.12)	3082 (33.12)	3214 (33.8)	3554 (34.82)	3836 (35.77)	4043 (37.19)	3869 (35.93)	32853 (34.3)
LOSH, mean (SD)*	9 (12)	9 (11)	8 (11)	8 (10)	8 (10)	8 (10)	8 (9)	7 (9)	7 (8)	7 (8)	8 (10)
IHM, n (%)	996 (12.21)	1019 (12.15)	1046 (11.81)	1041 (11.56)	1087 (11.68)	1081 (11.37)	1149 (11.26)	1183 (11.03)	1212 (11.15)	1234 (11.46)	11048 (11.53)
Diabetes											
N	1196	1372	1533	1619	1790	2055	2211	2315	2506	2635	19232
Incidence*	50.09	57.46	61.61	62.54	69.14	72.75	74.68	78.19	74.4	78.23	68.87
Age, mean (SD)*	74.37 (7.75)	73.53 (8.1)	73.96 (8.19)	74.61 (7.92)	74.51 (8.03)	74.71 (8.47)	75.03 (8.36)	74.88 (8.24)	75.38 (8.3)	75.34 (8.32)	74.75 (8.86)
Male, n (%)	1111 (92.89)	1293 (94.24)	1442 (94.06)	1482 (91.54)	1678 (93.74)	1917 (93.28)	2063 (93.31)	2163 (93.43)	2344 (93.54)	2448 (92.9)	17941 (93.29)
CCI 0–1, n (%)*	325 (27.17)	378 (27.55)	468 (30.53)	503 (31.07)	470 (26.26)	573 (27.88)	603 (27.27)	651 (28.12)	721 (28.77)	693 (26.3)	5385 (28.00)
CCI 2, n (%)	456 (38.13)	530 (38.63)	553 (36.07)	580 (35.82)	630 (35.2)	728 (35.43)	824 (37.27)	826 (35.68)	857 (34.2)	939 (35.64)	6923 (36.00)
CCI ≥3, n (%)	415 (34.7)	464 (33.82)	512 (33.4)	536 (33.11)	690 (38.55)	754 (36.69)	784 (35.46)	838 (36.2)	928 (37.03)	1003 (38.06)	6924 (36.00)
Smoking*	379 (31.69)	482 (35.13)	522 (34.05)	518 (32)	569 (31.79)	650 (31.63)	760 (34.37)	774 (33.43)	867 (34.6)	939 (35.64)	6460 (33.59)
LOSH, mean (SD)*	9 (11)	8 (11)	8 (11)	8 (10)	8 (11)	8 (10)	8 (10)	7 (9)	7 (8)	7 (8)	8 (10)
IHM, n (%)	129 (10.79)	137 (9.99)	149 (9.72)	161 (9.94)	172 (9.61)	194 (9.44)	192 (8.68)	201 (8.68)	236 (9.42)	231 (8.77)	1802 (9.37)

N: Number of discharges; Incidence: per 100,000 inhabitants; LOHS: length of stay; IHM: In-hospital mortality; CCI (Charlson Comorbidity Index): Comorbidities included in the Charlson comorbidity index, except diabetes.*P < 0.05 (ANOVA or Kruskal-Wallis analysis) or (*χ*2 linear trend analysis).

For both groups studied a significant increase in the mean age and in the prevalence of smoking was observed along the study period.

The mean LOHS fell from 9 days in 2003 to 7 days in 2012 for either patients with type 2 diabetes and in those without diabetes (p < 0.05).

Crude IHM remained stable overtime for both groups compared with patients with type 2 diabetes showing lower IHM than patients without diabetes in all years studied.

From 2003 to 2012, a total of 25,823 admissions of patients who underwent scheduled or unscheduled AAA repair procedures were recorded in Spain.

Tables 2 and 3 show the annual hospital discharges rates, clinical characteristic and outcomes for patients with open and endovascular repair procedures according to diabetes status from 2003 to 2012.

**Table 2:** **Incidences, clinical characteristics and outcomes of hospital discharges after open abdominal aortic aneurysm repair among patients with and without type 2 diabetes in Spain, 2003-2012**

	**2003**	**2004**	**2005**	**2006**	**2007**	**2008**	**2009**	**2010**	**2011**	**2012**	**Total**
No diabetes											
N	1447	1431	1458	1462	1365	1332	1329	1315	1177	1120	13436
Incidence*	12,28	12.15	11.94	11.56	10.8	10.26	9.98	9.87	8.6	8.18	10.49
Age, mean (SD)	70.03 (7.62)	69.91 (7.51)	70.02 (7.67)	69.83 (7.72)	69.92 (7.7)	70.04 (7.95)	69.71 (8)	69.52 (8.28)	69.54 (8.03)	69.97 (8.16)	69.85 (7.85)
Male, n (%)*	1377 (95.16)	1379 (96.37)	1407 (96.5)	1401 (95.83)	1292 (94.65)	1268 (95.2)	1240 (93.3)	1247 (94.83)	1094 (92.95)	1057 (94.38)	12762 (94.98)
CCI 0–1, n (%)*	826 (57.08)	773 (54.02)	832 (57.06)	867 (59.3)	811 (59.41)	761 (57.13)	770 (57.94)	762 (57.95)	721 (61.26)	704 (62.86)	7827 (58.25)
CCI 2, n (%)	448 (30.96)	485 (33.89)	446 (30.59)	457 (31.26)	408 (29.89)	430 (32.28)	426 (32.05)	428 (32.55)	343 (29.14)	311 (27.77)	4182 (31.13)
CCI ≥3, n (%)	173 (11.96)	173 (12.09)	180 (12.35)	138 (9.44)	146 (10.7)	141 (10.59)	133 (10.01)	125 (9.51)	113 (9.6)	105 (9.38)	1427 (10.62)
Smoking*	588 (40.64)	600 (41.93)	607 (41.63)	564 (38.58)	537 (39.34)	523 (39.26)	548 (41.23)	575 (43.73)	527 (44.77)	475 (42.41)	5544 (41.26)
LOSH, mean (SD)*	15 (15)	14 (15)	13 (15)	12 (13)	12 (14)	12 (14)	12 (13)	11 (12)	10 (12)	10 (11)	12 (14)
IHM, n (%)	272 (18.8)	259 (18.1)	231 (15.84)	236 (16.14)	241 (17.66)	223 (16.74)	226 (17.01)	219 (16.65)	205 (17.42)	183 (16.34)	2295 (17.08)
Diabetes											
N	160	205	180	196	208	203	212	188	234	216	2002
Incidence*	6.7	8.59	7.23	7.57	8.03	7.19	7.16	6.35	6.95	6.41	7.17
Age, mean (SD)	69.87 (6.57)	70.32 (7.27)	70.33 (6.97)	69.72 (7.7)	69.32 (7.55)	69.2 (7.41)	69.36 (7.95)	70.01 (7.4)	70.02 (8.08)	69.31 (8.11)	69.73 (7.55)
Male, n (%)	157 (98.13)	200 (97.56)	173 (96.11)	189 (96.43)	200 (96.15)	194 (95.57)	206 (97.17)	180 (95.74)	221 (94.44)	207 (95.83)	1927 (96.25)
CCI 0–1, n (%)	80 (50)	100 (48.78)	103 (57.22)	111 (56.63)	116 (55.77)	111 (54.68)	119 (56.13)	121 (64.36)	135 (57.69)	120 (55.56)	1116 (55.74)
CCI 2, n (%)	52 (32.5)	73 (35.61)	56 (31.11)	64 (32.65)	69 (33.17)	66 (32.51)	74 (34.91)	53 (28.19)	81 (34.62)	74 (34.26)	662 (33.07)
CCI ≥3, n (%)	28 (17.5)	32 (15.61)	21 (11.6)	21 (10.71)	23 (11.06)	26 (12.81)	19 (8.96)	14 (7.45)	18 (7.69)	22 (10.19)	224 (11.19)
Smoking	73 (45.63)	89 (43.41)	82 (45.56)	76 (38.78)	78 (37.5)	80 (39.41)	103 (48.58)	85 (45.21)	93 (39.74)	103 (47.69)	862 (43.06)
LOSH, mean (SD)*	16 (18.5)	13 (16)	12 (14)	13 (14)	12 (13)	11 (10)	11 (11.5)	11 (10)	9 (10)	9 (7)	11 (12)
IHM, n (%)	19 (11.88)	28 (13.66)	27 (15)	36 (18.37)	24 (11.54)	26 (12.81)	21 (9.91)	21 (11.17)	37 (15.81)	27 (12.5)	266 (13.29)

N: Number of procedures; Incidence: per 100,000 inhabitants; LOHS: length of stay; IHM: In-hospital mortality; CCI (Charlson Comorbidity Index): Comorbidities included in the Charlson comorbidity index, except diabetes.*P < 0.05 (ANOVA or Kruskal-Wallis analysis) or (*χ*2 linear trend analysis).

**Table 3:** **Incidences, clinical characteristics and outcomes of hospital discharges after endovascular abdominal aortic aneurysm repair among patients with and without type 2 diabetes in Spain, 2003-2012**

	**2003**	**2004**	**2005**	**2006**	**2007**	**2008**	**2009**	**2010**	**2011**	**2012**	**Total**
No diabetes											
N	424	479	589	652	766	835	1119	1257	1334	1379	8834
Incidence*	4.07	4.82	5.16	6.06	6.43	8.4	9.44	9.75	10.07	7.12	6.9
Age, mean* (SD)	72.08 (7.97)	73.11 (7.75)	72.55 (8.02)	73.31 (7.51)	73.56 (8.15)	73.58 (7.74)	73.65 (7.94)	73.99 (7.86)	73.85 (7.75)	73.68 (7.7)	73.51 (7.84)
Male, n (%)*	410 (96.7)	458 (95.62)	572 (97.11)	631 (96.78)	725 (94.65)	792 (94.85)	1052 (94.01)	1191 (94.75)	1261 (94.53)	1299 (94.2)	8391 (94.99)
CCI 0–1, n (%)	250 (58.96)	240 (50.1)	279 (47.37)	348 (53.37)	398 (51.96)	417 (49.94)	588 (52.55)	635 (50.52)	718 (53.82)	732 (53.08)	4605 (52.13)
CCI 2, n (%)	131 (30.9)	163 (34.03)	212 (35.99)	216 (33.13)	263 (34.33)	303 (36.29)	383 (34.23)	455 (36.2)	451 (33.81)	453 (32.85)	3030 (34.30)
CCI ≥3, n (%)	43 (10.14)	76 (15.87)	98 (16.64)	88 (13.5)	105 (13.71)	115 (13.77)	148 (13.23)	167 (13.29)	165 (12.37)	194 (14.07)	1190 (13.57)
Smoking*	151 (35.61)	192 (40.08)	222 (37.69)	246 (37.73)	288 (37.6)	346 (41.44)	451 (40.3)	528 (42)	562 (42.13)	611 (44.31)	3597 (40.72)
LOSH, mean (SD)*	8 (10)	8 (9)	8 (10)	8 (7.5)	8 (9)	7 (8)	7 (9)	7 (7)	6 (6)	6 (6)	7 (8)
IHM, n (%)	25 (5.9)	38 (7.93)	40 (6.79)	49 (7.52)	62 (8.09)	59 (7.07)	69 (6.17)	89 (7.08)	87 (6.52)	83 (6.02)	601 (6.80)
Diabetes											
N	51	63	86	104	109	166	209	262	258	243	1551
Incidence*	2.14	2.64	3.46	4.02	4.21	5.88	7.06	8.85	7.66	7.21	5.55
Age, mean* (SD)	72.88 (6.51)	72.37 (7.79)	72.36 (7.21)	73.7 (6.61)	72.39 (7.52)	72.82 (7.54)	73.24 (7.23)	72.98 (7.34)	73.69 (7.65)	73.16 (7.65)	73.09 (7.39)
Male, n (%)	49 (96.08)	59 (93.65)	86	103 (99.04)	104 (95.41)	161 (96.99)	203 (97.13)	257 (98.09)	247 (95.74)	233 (95.88)	1502 (96.84)
CCI 0–1, n (%)*	21 (41.18)	21 (33.33)	41 (47.67)	41 (39.42)	47 (43.12)	70 (42.17)	89 (42.58)	138 (52.67)	124 (48.06)	130 (53.5)	722 (46.55)
CCI 2, n (%)	26 (50.98)	30 (47.62)	28 (32.56)	42 (40.38)	38 (34.86)	59 (35.54)	91 (43.54)	95 (36.26)	97 (37.6)	75 (30.86)	581 (37.46)
CCI ≥3, n (%)	4 (7.84)	12 (19.05)	17 (19.77)	21 (20.19)	24 (22.02)	37 (22.29)	29 (13.88)	29 (11.07)	37 (14.34)	38 (15.64)	248 (15.99)
Smoking*	18 (35.29)	33 (52.38)	34 (39.53)	37 (35.58)	34 (31.19)	59 (35.54)	100 (47.85)	120 (45.8)	109 (42.25)	106 (43.62)	650 (41.91)
LOSH, mean (SD)	9 (8)	7 (7)	8 (7)	7 (8)	8 (12)	7 (6)	7 (6)	7 (8)	7 (7)	6 (6)	7 (7)
IHM, n (%)	3 (5.88)	6 (9.52)	3 (3.49)	9 (8.65)	9 (8.26)	11 (6.63)	12 (5.74)	11 (4.2)	15 (5.81)	12 (4.94)	91 (5.87)

N: Number of procedures; Incidence: per 100,000 inhabitants; LOHS: length of stay; IHM: In-hospital mortality; CCI (Charlson Comorbidity Index): Comorbidities included in the Charlson comorbidity index, except diabetes.*P < 0.05 (ANOVA or Kruskal-Wallis analysis) or (*χ*2 linear trend analysis).

Over the study period, 13.7% (n = 3,553) of all patients underwent AAA repair procedures had type 2 diabetes. There were 15,438 open AAA repair procedures (12.9% [n = 2,002] in patients with type 2 diabetes) and 10,385 endovascular AAA repair procedures (14.9% [n = 1,551] in patients with type 2 diabetes).

In patients who underwent an OSR, there was a significant male predominance in patients both with and without diabetes (96.3% and 95%). Mean age was 69.73 years (SD, 7.55 years) in patients with type 2 diabetes and 69.85 years (SD, 7.85 years) in those without diabetes.

Patients with type 2 diabetes who underwent OSR procedure had significantly higher CCI values compared to those without diabetes (44.3% vs. 41.8% with two or more coexisting conditions, respectively).

Among those who underwent OSR, the mean LOHS was 11 days in patients with type 2 diabetes and 12 days in those without diabetes. Also, IHM was 13.3% in patients with type 2 diabetes and 17.1% in patients without diabetes.

As can be seen in Table 2, among patients with type 2 diabetes who underwent open AAA repair, the incidence of discharges decreases significantly from 6.7 cases per 100,000 inhabitants in 2003 to 6.41 cases in 2012. In patients without diabetes, the incidence decreased from 12.28 cases per 100,000 inhabitants in 2003 to 8.18 cases in 2012 (p < 0.05).

We found that the proportion of men with diabetes who underwent OSR decreased from 98.1% in 2003 to 95.8% in 2012 and the prevalence of those with CCI of two or more fell from 50% in 2003 and 44. 5% in 2012.

LOHS after OSR decreased significantly over the study period in both groups of patients (16 days in 2003 vs. 9 days in 2012, in patients with type 2 diabetes and 15 days in 2003 vs. 10 days in 2012, in those without diabetes).

The IHM among those who underwent an OSR did not change significantly for those with and without type 2 diabetes. We found that over the entire period, in patients who underwent an EVAR, the mean age was 73.09 years (SD, 7,39 years) in patients with type 2 diabetes and 73.51 years (SD, 7,84 years) in those without diabetes and there was higher proportion of males undergoing endovascular AAA procedures in both groups (96.8% in patients with type 2 diabetes and 95% in patients without diabetes).

In our study, patients with diabetes who underwent an EVAR procedure had significantly higher CCI values than those without diabetes (53.5% vs. 47.9% with two or more comorbidities). In patients with type 2 diabetes, the overall IHM was 5.9% and in those without diabetes 6.8%; however the mean LOHS was 7 days in both groups.

As can be seen in Table 3 among patients with type 2 diabetes who underwent an EVAR the incidence of discharges increased significantly from 2.14 cases per 100,000 inhabitants in 2003 to 7.21 cases in 2012. In patients without diabetes, the incidence raised significantly from 4.07 cases per 100,000 inhabitants in 2003 to 7.12 cases in 2012.

Over the 10-year study period, the mean age of patients with type 2 diabetes who underwent an EVAR increased from 72.88 years (SD, 6.51 years) in 2003 to 73.16 years (SD, 7.65 years) in 2012 (p < 0,05).

In our study, LOSH in patients without diabetes decreased significantly from 8 days (SD, 10 days) in 2003 to 6 days (SD, 6 days) in 2012, but remained stable in those with type 2 diabetes over the period of study (Table 3). The IHM among those who underwent an EVAR remained stable for those with and without type 2 diabetes during the 10-year study.

Table 4 summaries the results of multivariate analysis of trends and factors associated with incidence and IHM among patients with and without diabetes hospitalized for AAA. From 2003 to 2012, the adjusted incidence rate ratio of having a AAA discharge diagnosis in patients with type 2 diabetes was significant and higher than in those without diabetes(1.09 95%CI 1.08-1.09 vs. 1.03 95% CI 1.02-1.04).

**Table 4:** **Multivariate analysis of the factors associated with incidence and mortality due to abdominal aortic aneurysm among patients with and without type 2 diabetes in Spain, 2003-2012**

		Incidence (IRR)*			**In-hospital mortality (OR)‡**		
		**Diabetes**	**No Diabetes**	**Total**	**Diabetes**	**No Diabetes**	**Total**
Sex	Men	1	1	1	1	1	1
	Female	0.08 (0.07-0.09)	0.09 (0.08-0.010)	0.09 (0.08-0.010)	1.38 (1.17-1.65)	1.16 (1.08-1.24)	1.19 (1.12-1.27)
Age groups (years)	50-59	1	1	1	1	1	1
	60-69	4.07 (3.78-4.38)	3.69 (3.58-3.80)	3.74 (3.64-3.85)	1.13 (0.83-1.54)	1.09 (0.97-1.23)	1.09 (0.98-1.22)
	70-79	9.10 (8.50-9.74)	7.56 (7.35-7.78)	7.79 (7.58-7.99)	1.55 (1.16-2.07)	1.59 (1.42-1.77)	1.58 (1.43-1.75)
	≥80	5.93 (5.54-6.37)	5.79 (5.62-5.96)	5.80 (5.65-5.96)	2.71 (2.02-3.64)	2.83 (2.53-3.16)	2.81 (2.53-3.12)
Charlson index	0-1	1	1	1	1	1	1
	2	1.29 (1.24-1.34)	1.03 (1.01-1.05)	1.07 (1.05-1.08)	1.36 (1.18-1.56)	1.03 (0.98-1.09)	1.07 (1.02-1.13)
	≥3	1.30 (1.26-1.36)	0.91 (0.89-0.93)	0.96 (0.95-0.98)	2.12 (1.93-2.53)	1.68 (1.59-1.77)	1.74 (1.65-1.82)
Smoking	No	1	1	1	1	1	1
	Yes	0.53 (0.51-0.55)	0.52 (0.51-0.53)	0.53 (0.52-0.54)	0.65 (0.57-0.73)	0.60 (0.57-0.62)	0.60 (0.58-0.63)
Open abdominal aortic aneurysm repair	No	1	1	1	1	1	1
	Yes	0.13 (0.12-0.14)	0.17 (0.16-0.17)	0.16 (0.15-0.18)	2.52 (2.16-2.93)	2.55 (2.41-2.69)	2.55 (2.42-2.68)
Endovascular aortic aneurysm repair	No	1	1	1	1	1	1
	Yes	0.10 (0.9-0.101	0.10 (0.9-0.11)	0.10 (0.9-0.12)	0.82 (0.65-1.02)	0.71 (0.65-0.77)	0.72 (0.67-0.78)
Diabetes	No	NA	NA	1	NA	NA	1
	Yes	NA	NA	0.20 (0.19-0.21)	NA	NA	0.81 (0.76-0.85)
Year		1.09 (1.08-1.09)	1.03 (1.02-1.04)	1.04 (1.03-1.05)	0.98 (0.96-0.99)	0.98 (0.97-0.99)	0.98 (0.97-0.99)

*Calculated using multivariate Poisson regression: Incidence Rate Ratios (IRR). ‡ Calculated using logistic regression models: Odds Ratio (OR). The logistic regression multivariate model and Poisson regression model were built using as dependent variables “death (yes/no)” and “incidence” respectively, and as independent variables year, sex, Charlson comorbidity index, and age.

Incidence rate ratios were significantly greater in older subjects, especially those age 70–79 years, and patients with ≥2 comorbidities for those with and without diabetes.

With regard to IHM, among subjects with diabetes IHM was significantly greater in women than in men (OR, 1.38; 95%CI, 1.17-1.65), in older subjects (OR 2.71; 95%CI 2.02-3.64 in ≥80 aged group compared with reference category 50–59 years) and in those with more comorbidities (OR 1.36; 95%CI, 1.18-1.56 for those with 2 comorbidities and OR 2.12; 95%CI, 1.93-2.53 for those with ≥3 comorbidities). Over the entire period of time and after adjusting the model for the rest of the variables, the probability of dying of a diabetic smoker patient with AAA is 0.65 (95%CI 0.57-0.73) times lower than for a diabetic non smoker patient.

Those diabetic patients who received an OSR procedure had a 2.52-fold (95%CI 2.06-2.93) higher probability of dying during their stay than those who did not undergo this procedure. Time trend analysis showed a significant decrease in mortality from 2003 to 2012 (OR 0.98 95% CI 0.96-0.99. As can be seen in Table 4 the same variables were associated to IHM among those without diabetes.

When we analyzed the entire database, patients with type 2 diabetes had significantly lower mortality than patients without diabetes after adjusting for all covariates (OR, 0.81; 95%CI, 0.76-0.85).

## Discussion

Our results reveal that more than 16% of Spanish adults who suffer AAA have an associated diagnosis of diabetes. These results are consistent with those of De Rango *et al*. (2012), who showed that 13.6% of patients admitted to hospital for AAA in Italy had diabetes [25].

Using the Spanish National Hospital Database, we found that rates of hospitalization for AAA in patients with and without type 2 diabetes increased significantly from 2003 to 2012, but incidence rates were higher in non-diabetic patients over the period of study. In Italy, Sensi *et al*. indicated that when they focused on elderly patients (aged 75 years or more) hospitalization rates increased over time and explain this trend as a delayed clinical onset of illness, resulting from the combined effect of genetic factors and the decrease of smoking [26]. In US, Mureebe *et al*. found a significant increase in the number of hospitalizations for intact AAA from 296 per 100,000 in 1995 to 341 per 100,000 beneficiaries in 2006. They indicated that AAA rates have increased significantly due to elective repair of large aneurysm prevents subsequent rupture and another potential explanation for the increase in number of admissions for the diagnosis of AAA was that there has been for patients who have EVAR, and those patients may be inflating the admission pool of those patients with the diagnosis [27].

After adjusting by age and sex the incidence of hospital discharge with an AAA diagnosis was significantly lower among men and women with diabetes as compared to non diabetic subjects.

Previous reports based in cross-sectional studies of AAA screening and case–control studies have shown an inverse association between diabetes and AAA [3,5,28,29]. In 2015, Shah *et al*. investigated this evidence from large prospective cohort studies with 3113 AAA events and concluded that type 2 diabetes was inversely associated with AAA (adjusted HR 0.46 95%IC 0.35-0.59) [4].

We found an increase in EVAR procedure rates and decline in hospital admissions for OSR in patients with and without type 2 diabetes from 2003 to 2012. Sensi *et al*. indicated that EVAR technique became the preferred method for AAAs since 2008 and the advantage of this surgical procedure is that it requires shorter hospitalizations, although is more costly [26].

Our investigation reinforces the well-known fact that AAA incidence is significantly lower in women than in men [3]. However, despite having one fifth the number of AAAs than men, women constitute approximately one-third of all ruptures and almost as many deaths as men [30,31]. In our study IHM was significantly greater in women with than in men with or without diabetes. Mureebe *et al*. (2010) found that female gender was associated with increased risk of death (OR 1.53; CI95% 1.47-1.58) in patients with ruptured AAA and the mortality rate for women was higher by 8.9% for open repair and higher by 7.1% for EVAR vs. men. These authors suggest that biologic factors and mechanical properties of the aorta may contribute to the differences seen between men and women [32].

In our study, the IHM decreased over time among diabetic and non-diabetic patients with a diagnosis of AAA. However because the number of hospitalizations increased over time, the decreased in mortality rates suggest good quality of care [26].

Patients with type 2 diabetes had significantly lower mortality than patients without diabetes. One explanation is the presence of diabetes might be an additional factor to select the best strategy to apply in individual small AAAs. It has been shown that for patients with small AAA under surveillance, the risk of death, aneurysm-related death, and aneurysm rupture are very low (less than 1% per year) and, therefore, surveillance has been largely recognized as the best strategy for most patients [25].

Another explanation could be the “obesity paradox” [33]. An elevated body mass index (BMI) may be associated with a decrease in AAA mortality. It is well known that obesity is much more frequent among those with than without diabetes. In 2014 Sidloff *et al*. concluded that BMI demonstrated a negative linear association with AAA mortality (P < 0.007) [34]. A recent prospective study of 18782 persons with diagnosis of AAA in US concluded that a BMI ≥25 kg/m2 was protective (HR 0.72; 95% CI 0.53-0.98) [35].

Different studies suggest that it is hyperglycemia that retards aneurysm progression and mortality by AAA. The Health in Men study reported a negative association between fasting glucose and aortic diameter in 2859 non-diabetics [36]. Investigators at Standford reported that hyperglycemia in mice was associated with slower AAA enlargement, and this effect was diminished by insulin therapy [37].

In our investigation smoking was associated to significantly lower in hospital mortality for patients with and without diabetes.. Kent *et al*. concluded that a substantial number of women, nonsmokers, and people < 65 with multiple comorbities have a risk of AAA equivalent to or greater than that of 65-year-old male ever-smokers [3]. The survival effect, meaning that those who even suffering diabetes continue smoking are those with less severe disease and less comorbid conditions, may explain this association. Also different coding for those who die in the hospital and those who survive could explain this association. In any case further studies are necessary to clarify this association.

We found that patients who received an OSR procedure had higher probability of dying during their stay than those who did not undergo this procedure. Our results are consistent with reported in the literature indicating higher 30-day mortality for OSR [38-40].

A recent metaanalysis indicated operative mortality (30-day/in-hospital) after AAA treatment is increased in diabetics (OR 1.26 95%CI 1.10-1.44) but IHM after repair in diabetic patients showed no significant higher risk in these patients (OR 0.97 95%CI 0.63-1.50) [3].

Mureebe *et al*. (2008) indicated that when they evaluated the effect of EVAR on death, their results agreed with other studies that found a beneficial effect of EVAR in ruptured AAA and concluded that increased usage of this technology will likely sustain continuous improvement in survival in the future [27].

The strength of our investigation lies in its large sample size, its 10-year follow-up period and its standardized methodology, which has previously been used to investigate diabetes and its complications in Spain and elsewhere [41,42]. Nevertheless, our study is subject to a series of limitations. Our data source was the CMBD, an administrative database that contains discharge data for Spanish hospitalizations and uses information the physician has included in the discharge report; therefore, a limitation important to the analysis of the current data set is the lack of information about patient anatomy. We are not able to identify which of these patients had diseased iliac arteries, nor do we know the size of the aneurysms treated. Another significant limitation is the fact that we have not broken down our diabetic patients into groups based on therapy used to control blood glucose, and we were unable to state their blood glucose control pre- and postoperatively.

The present study also lacks information regarding the specific effects of diabetic medication on AAA. De Rango *et al.* indicated that data supporting the protective effect of hypoglycemic or other medication against AAA (e.g., statins, fibrates) are limited and require more in-depth analysis of their true efficacy [25].

Another limitation of this database is its anonymity (no identifying items such as clinical history number), which makes it impossible to detect whether the same patient was admitted more than once during the same year. In addition, patients who moved from one hospital to another would appear twice.

Nevertheless, this dataset, which was introduced in Spain in 1982, is a mandatory register, and its coverage is estimated to be greater than 95% [19]. Concerns have been raised about the accuracy of routinely-collected datasets; however, these datasets are periodically audited. Consequently, the quality and validity of our dataset has been assessed and shown to be useful for health research [43].

## Conclusions

Our results show that rates of hospitalization for AAA in patients with and without type 2 diabetes increased significantly from 2003 to 2012, but incidence rates were higher in non-diabetic patients over the period of study for both sexes and patients aged over 70 years. Patients with type 2 diabetes had significantly lower mortality than patients without diabetes. Higher comorbidity and female gender in patients with AAA are associated with higher IHM for both groups of patients.

We found a decrease in the use of OSR procedures and an increase in the use of EVAR procedures in patients with and without type 2 diabetes. Patients who received an OSR procedure had higher probability of dying during their stay than those who did not undergo this procedure.

Given the rapid increase in the prevalence of diabetes and the aging population, these findings emphasize the need for further improvement in the control of AAA risk factors in people with diabetes.

## Abbreviations

AAA: Abdominal aortic aneurysm
CCI: Charlson comorbidity index
CMBD: Spanish minimum basic data set (*Conjunto Mínimo Básico de Datos)*
EVAR: Endovascular aneurism repair
ICD-9-CM: International classification diseases-ninth revision, Clinical modification
IHM: In-hospital mortality
LOHS: Length of hospital stay
OSR: Open surgery repair
