# Erratum to: National trends in incidence and outcomes of abdominal aortic aneurysm among elderly type 2 diabetic and non-diabetic patients in Spain (2003–2012)

**DOI:** 10.1186/s12933-016-0423-4

**Published:** 2016-07-21

**Authors:** Ana Lopez-de-Andrés, Isabel Jiménez-Trujillo, Rodrigo Jiménez-García, Valentín Hernández-Barrera, José Mª de Miguel-Yanes, Manuel Méndez-Bailón, Napoleón Perez-Farinos, Miguel Ángel Salinero-Fort, Pilar Carrasco-Garrido

**Affiliations:** Preventive Medicine and Public Health Teaching and Research Unit, Health Sciences Faculty, Rey Juan Carlos University, Avda. de Atenas s/n, 28922, Alcorcon, Comunidad de Madrid Spain; Medicine Department, Hospital Gregorio Marañon, Madrid, Comunidad de Madrid Spain; Medicine Department, Hospital Clínico San Carlos, Madrid, Comunidad de Madrid Spain; Health Security Agency, Ministry of Health, Madrid, Comunidad de Madrid Spain; Dirección Técnica de Docencia e Investigación, Gerencia Atención Primaria, Madrid, Comunidad de Madrid Spain

## Erratum to: Cardiovascular Diabetology (2015) 14:48 DOI 10.1186/s12933-015-0216-1

After publication of the original article [1], it came to the authors’ attention that a source of funding for the study presented had been inadvertently omitted. The Acknowledgements section should therefore have read as follows:

“This study was funded by the FIS (*Fondo de Investigaciones Sanitarias*—Health Research Fund, Grant No. PI13/00118, *Instituto de Salud Carlos III*), co-financed by the European Union through the Fondo Europeo de Desarrollo Regional (FEDER, “*Una manera de hacer Europa*”), and by the *Grupo de Excelencia Investigadora URJC*-*Banco Santander* No. 30VCPIGI03: *Investigación traslacional en el proceso de salud*—*enfermedad* (ITPSE).”
